# A Delphi study to explore clinician and lived experience perspectives on setting priorities in eating disorder services

**DOI:** 10.1186/s12913-022-08170-4

**Published:** 2022-06-17

**Authors:** Katie L. Richards, Isabel Woolrych, Karina L. Allen, Ulrike Schmidt

**Affiliations:** 1 Department of Psychological Medicine, Institute of Psychiatry, Psychology and Neuroscience, King’s College London, London, UK; 2 Eating Disorder Outpatient Service, South London and Maudsley NHS Foundation Trust, London, UK

**Keywords:** Eating disorders, Priority setting, Delphi study, Waiting lists

## Abstract

**Background:**

Due to scarce resources and high demand, priority setting in mental health services is necessary and inevitable. To date, no study has examined priority setting in eating disorder (ED) services specifically. Here, we evaluate the level of consensus and perceived relative importance of factors used to determine patient prioritisation in ED services, amongst clinicians and individuals with lived experience (LE) of an ED.

**Methods:**

A three round Delphi study and a ranking task were used to determine the level of consensus and importance. Consensus was defined as > 80% agreement or disagreement. Items that reached consensus for agreement were ranked in order of importance from most to least important. Participants were 50 ED clinicians and 60 LE individuals. Participant retention across rounds 2, 3, and 4 were 92%, 85%, and 79%, respectively.

**Results:**

Over three iterative rounds, a total of 87 statements about patient prioritisation were rated on a 5-point Likert-scale of agreement. Twenty-three items reached consensus in the clinician panel and 20 items reached consensus in the LE panel. The pattern of responding was broadly similar across the panels. The three most important items in both panels were medical risk, overall severity, and physical health deteriorating quickly. Clinicians tended to place greater emphasis on physical risk and early intervention whereas the LE panel focused more on mental health and quality of life.

**Conclusions:**

Eating disorder services tend to prioritise patients based upon medical risk and severity, and then by the order in which patients are referred. Our findings align in some respects with what is observed in services, but diverge in others (e.g., prioritising on quality of life), providing important novel insights into clinician and LE opinions on waiting list prioritisation in EDs. More research is warranted to validate these findings using multi-criterion decision techniques and observational methods. We hope these findings provide a foundation for future research and encourage evidence-based conversations around priority setting in ED services.

**Supplementary Information:**

The online version contains supplementary material available at 10.1186/s12913-022-08170-4.

Waiting lists and their management are a major issue for publicly funded mental health services [1]. Waiting can increase distress, risk, and negatively impact outcomes and functioning [2–4]. Several initiatives have been proposed to manage waiting lists including wait time targets, and structured prioritisation tools and procedures [5–7]. In England, wait-time targets were introduced in 2016 for early intervention in psychosis and child and adolescent eating disorder (ED) services. These targets, alongside additional funding and performance monitoring, led to substantially improvements in rapid access to care [6, 8]. There are now plans to introduce similar targets for all mental health services in England [9].

Despite such efforts, demand continues to exceed supply, making effective priority setting procedures necessary. There are, however, only a limited number of tools for priority setting in mental health, most of which are non-specific and for child and adolescent services [5, 10].

Eating disorders are serious, life-threatening illnesses that cause considerable distress and have long-term implications for physical, social, and occupational functioning [11]. The limited availability of specialist ED services in many countries, alongside the unique challenges presented by EDs (e.g., ambivalence, extreme physical risk), make ED patient prioritisation daunting, even for experienced clinicians. Prioritisation decisions can lead to ethical dilemmas where individuals are required to balance professional considerations and institutional constraints alongside personal and moral judgements about what is “right” [12, 13]. There are no explicit frameworks and limited research to support decision making for ED service prioritisation. A systematic search identified only one relevant study, where patients with either obesity or anorexia nervosa (AN) were prioritised based upon age, social class, and mental health history. Patients were more likely to be prioritised if they were younger, with a comorbid mental health problem and from a low social class [14].

Three ethical principles of distributive justice are frequently used to guide priority setting decisions in healthcare: egalitarianism, utilitarianism, and prioritarianism [15, 16]. Egalitarianism aims to reduce inequalities and equalise lifetime health across the population. It is based on the premise that everyone is equally deserving of a long and healthy life, and is associated with distributive mechanisms such as ‘first-come first-served’ or lottery allocation. The UK National Health Service (NHS) is fundamentally egalitarian, providing access to all regardless of disadvantage [17]. Utilitarianism aims to maximise the aggregate total benefit to the population by directing care to those that will benefit the most, often quantified using quality-adjusted life years. Finally, prioritarianism, which closely aligns with the ‘rule of rescue’ (the desire to save those facing death), gives priority to individuals who are the worst-off, sickest, or most in need of care [15, 16].

The National Institute for Health and Care Excellence [18] recommend that patients with EDs should be treated as soon as possible, especially individuals with or at risk of severe emaciation, suggesting a tendency towards prioritarianism. In line with this, ED services typically prioritise patients based upon clinical priority and urgency in the first instance (e.g., body mass index (BMI) < 15 kg/m^2^, rapid weight loss) followed by the order in which they were referred. Prioritarianism is widespread within healthcare and even without formal prioritisation policies, patients with more severe and disabling presentations tend to be seen quicker [19–21]. Recent early intervention initiatives in EDs are more utilitarian, as they advocate for prioritising patients in early-stage illness, where treatment can be quicker and more effective [22–26]; however, see [27]. Utility and health gain are consistently valued in priority setting studies, sometimes emerging as the most important attribute (e.g., [28–30]). However, the importance of utilitarianism varies by context and the degree of health gained [31, 32]. Moreover, utilitarian approaches create complex ethical dilemmas where individuals with chronic illnesses and disabilities risk being disadvantaged [33].

Balancing equity, efficiency, and prioritarian goals is a challenge for developing transparent and fair priority setting procedures and policies in healthcare [34]. No single distributive theory is likely to ensure healthcare resources are allocated justly. Multi-allocation systems are often needed alongside evidence of value systems endorsed by the communities affected by such decisions [16]. An evaluation of clinician and patient perspectives, i.e., the people who are most directly involved in and affected by wait list decisions, would provide some much-needed insights and currency for discussion for what is a very challenging issue faced by ED services. To the best of our knowledge, there are no priority setting studies assessing the views of ED clinicians or individuals with lived experience (LE) of an ED. Here, we describe a Delphi study in which the collective opinions of clinicians and individuals with LE were sought to evaluate the level of consensus (agreement/disagreement) and perceived relative importance of factors used to determine patient prioritisation in ED services. The Delphi method is particularly well-suited for areas where there is limited research, no set standard, and for determining collective community-based values to facilitate decision making [35].

## Methods

### Study design

The Delphi method is a systematic approach for determining the level of consensus or dissensus (widespread dissent) among ‘experts’ on a given topic. The term ‘expert’ refers to someone who has professional or personal experience and knowledge on a topic [36]. A Delphi study typically involves multiple iterative rounds of questionnaires whereby feedback on responses is provided and items are re-rated considering this feedback. Participants are anonymous and rate items independently. This technique allows participants to reflect on their own position, and answer/amend answers without pressure from domineering group members [37, 38].

### Participants

Participants were recruited online via social media platforms (Twitter, Facebook, Instagram), and professional organisations and networks (including the British Eating Disorder Society, First Episode Rapid Early Intervention for EDs Network, and Eating Disorder Specialist Interest Groups). Expertise was defined as: (1) a practicing healthcare professional with at least one year’s worth of experience in EDs for the clinician panel; or (2) a current or previous diagnosis of Diagnostic and Statistical Manual of Mental Disorders-5 ED for the LE panel. A total of 110 individuals (50 clinicians and 60 individuals with LE) took part in the study. The demographic characteristics are outlined in Table 1.

**Table 1:** Participant characteristics

	Clinician (*n* = 50)		Lived experience (*n* = 60)
Age in years (*M, SD*)	41.24 (10.47)	Age in years (*M, SD*)	29.78 (2.33)
Gender (*n*, %)		Gender (*n*, %)	
Female	41 (82)	Female	53 (88)
Male	9 (18)	Male	6 (10)
Non-binary	0	Non-binary	1 (2)
Ethnicity (*n*, %)		Ethnicity (*n*, %)	
White/White British	47 (94)	White/White British	56 (93)
Asian/Asian British	1 (2)	Asian/Asian British	3 (5)
Black/Black British	1 (2)	Black/Black British	0
Mixed/Multiple or other ethnic background	1 (2)	Mixed/Multiple or other ethnic background	1 (2)
Profession (*n*, %)		Diagnosis^a^ (*n*, %)	
Psychiatrist	9 (18)	Anorexia nervosa	48 (80)
Clinical Psychologist	9 (18)	Bulimia nervosa	12 (20)
Psychiatric nurse	14 (28)	Binge eating disorder	7 (12)
Psychotherapist	6 (12)	OSFED/Atypical/Purging disorder	21 (35)
Occupational therapist	4 (8)	ARFID	5 (8)
Dietician	1 (2)	Comorbid neurodevelopmental disorder	8 (13)
Other	7 (14)	Other comorbid disorder (including mood, anxiety, and personality disorder)	46 (77)
Years working in EDs (*n*, %)		Time since ED onset in years (*M, SD*)	11.48 (8.31)
< 4 years	16 (32)	Recovered (*n*, %)	
5–15 years	28 (56)	Yes	18 (30)
> 16 years	6 (12)	Partially	16 (27)
		No	24 (40)
		Unsure	2 (3)
Work settings^a^ (*n*, %)		Treatment setting^a^ (*n*, %)	
Inpatient	35 (70)	Inpatient	24 (40)
Day patient	20 (40)	Day patient	22 (37)
Outpatient	25 (50)	Outpatient	55 (92)
Public	48 (96)	Public	56 (93)
Private	11 (22)	Private	28 (47)
CAMHS/CAEDS	20 (40)	CAMHS/CAEDS	23 (38)
AMHS/AEDS	45 (90)	AMHS/AEDS	46 (77)
All-age service (0–25 years)	4 (8)	All-age service (0–25 years)	8 (13)

*Note. OSFED* other specified feeding and eating disorder, *ARFID* avoidant restrictive food intake disorder, *ED* eating disorder, *CAMHS* child and adolescent mental health service, *CAEDS* child and adolescent eating disorder service, *AMHS* adult mental health service, *AEDS* adult eating disorder service

^a^ Participants can endorse multiple categories

### Procedure

The study involved a three round Delphi (Round 1–3) and a ranking task (Round 4) distributed via the cloud-based online survey platform Qualtrics, Provo, UT, Version April-August 2021 [39]. A modified Delphi method was used for this study, where the first round consisted of structured statements rather than open-ended questions. Modified Delphi methods are frequently used to minimise participant burden or provide a *seed list* derived from the literature [35, 40–42]. For the current study, this approach was selected to ensure that opinions were gathered on specific clinical (e.g., duration of illness) and non-clinical (e.g., socio-economic status) factors identified as important for priority setting in EDs specifically and health care more broadly. This approach was also selected to reduce the number of rounds and therefore time commitment required to take part in the study. The questionnaire for Round 1 was developed by conducting a systematic literature review followed by consultation and pre-testing with ED clinicians and individuals with LE (see Additional File 1 for details). Data collection occurred between April and August 2021. Each round took place over a 4 to 6-week period. Participants remained anonymous to one another throughout the study.

**Fig. 1:**
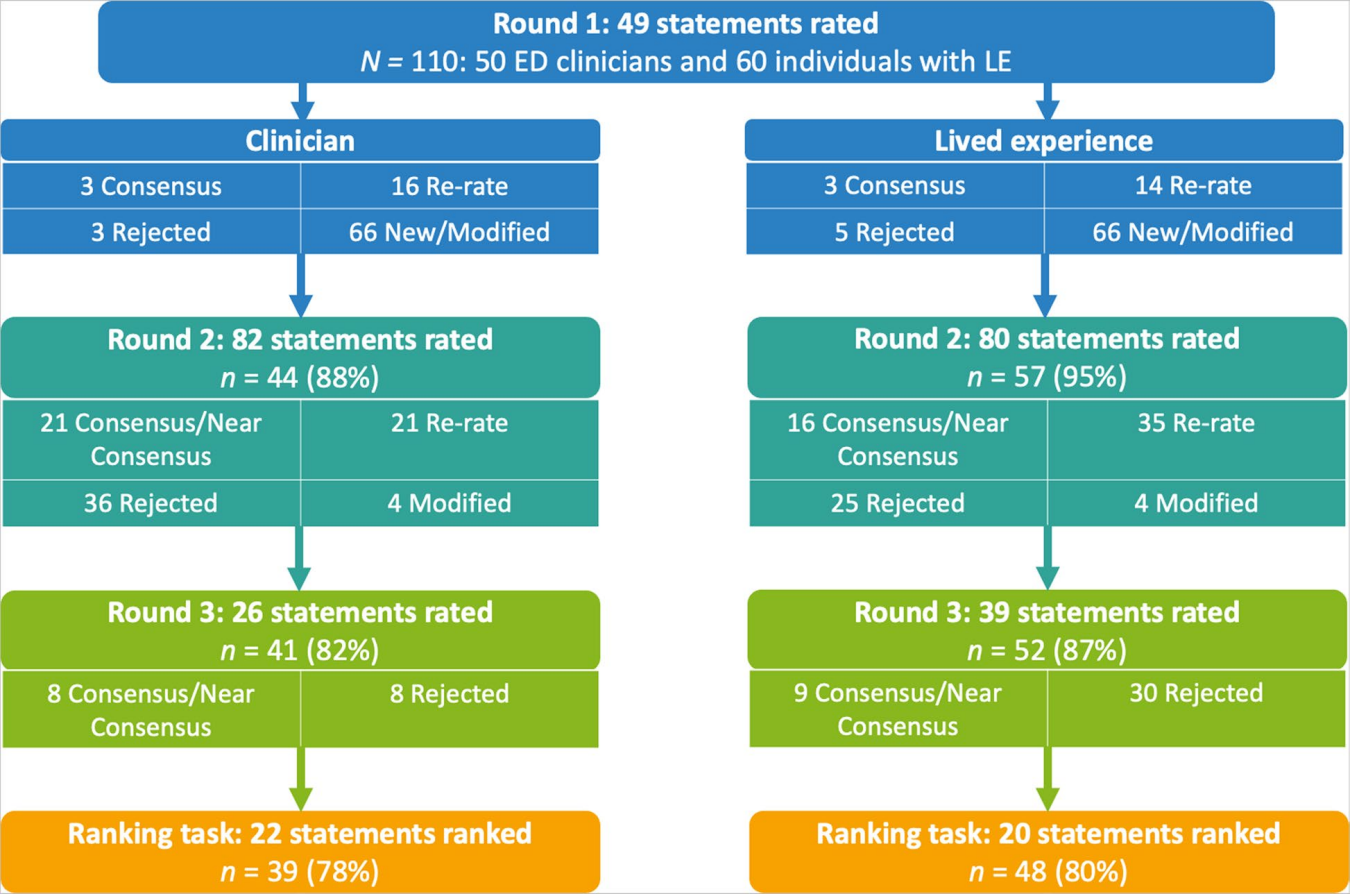
A flow chart of response rate, number items rated or ranked, and number of items that reached consensus/near consensus, or were re-rated, rejected, or new/modified per Delphi study round. *LE* lived experience, *ED* eating disorder

**Fig. 2:**
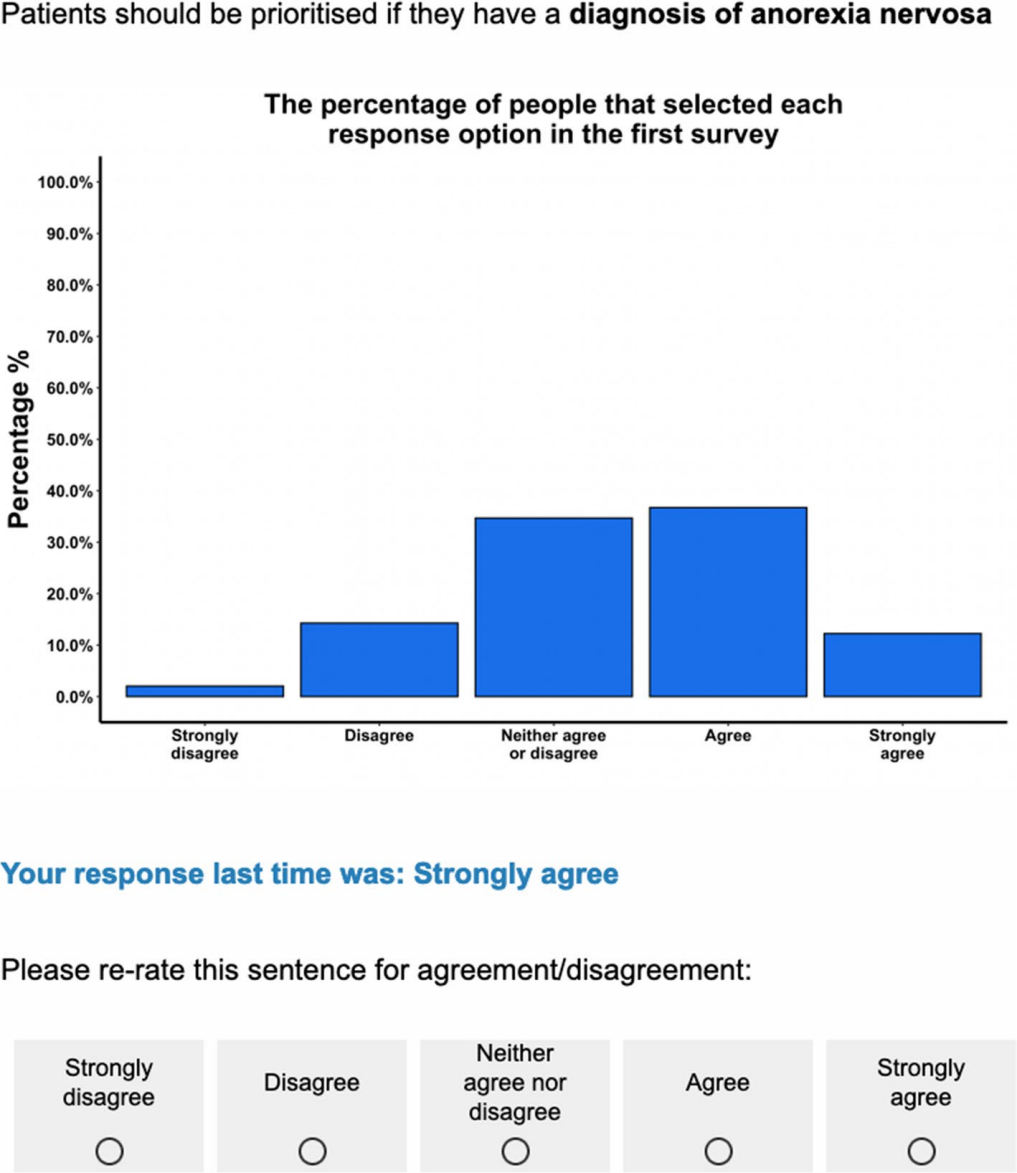
Example item and feedback from Round 2

Participants contacted the researchers (KR and IW) by email to express interest in taking part. Once eligibility was confirmed, a link to the consent form and first survey was provided. In Round 1–3, participants were presented with statements about patient prioritisation (e.g., “Patients should be prioritised if they have a diagnosis of anorexia nervosa”) and asked to rate each statement on a 5-point Likert scale ranging from ‘Strongly disagree’ (1) to ‘Strongly agree’ (5). Participants were asked to rate the items in relation to priority in ED services and for what their answer would be in most situations. The order of the statements was randomised for each participant. An optional comment box was provided alongside each statement where participants could provide feedback on language/wording, difficulties in understanding, or reasons why they gave a specific rating. The number of prioritisation statements per round are outlined in Fig. 1. In Round 1, an additional open-ended question was included at the end of the survey to identify new prioritisation factors. In Rounds 2 and 3, statements that were re-rated from previous rounds were accompanied by a histogram showing the distribution of responses and the participant’s own response from the previous round (see example in Fig. 2). Round 4 involved a ranking task, whereby participants were presented with the list of statements that reached consensus for agreement for their panel. Participants were asked to select the 10 most important items and rank them in order of importance from most to least important. Participants could also provide feedback on the ranking task in an optional comment box.

### Analysis

The qualitative responses for each round were independently analysed by two of the study authors (KR and IW) using an inductive content analysis method [43]. Open coding was used to identify new prioritisation factors and issues in questionnaire completion. The coding was completed in NVivo (Version 12) [44]. The results of the independent coding process were discussed by the two researchers who conducted the coding (KR and IW). During these discussions, the coders compared and contrasted codes to identify similarities and differences, and based upon these discussions added, modified, or removed items from each survey accordingly. All modifications and new items were integrated into clinician and LE surveys regardless of which group the qualitative feedback came from. The other study authors (KA and US) provided feedback on proposed changes and resolved discrepancies between the coders. The number of modified or new items per round are outlined in Fig. 1.

Frequencies were calculated in SPSS (version 27) and used to determine the percentage of consensus for each item. Consensus was calculated separately for each panel. In Rounds 1 and 2, items were sorted into three categories: ‘consensus’, ‘re-rate’, and ‘rejected’. ‘Consensus’ and ‘rejected’ items were removed and ‘re-rate’ items were re-administered. Consensus was defined as items that obtained ≥ 80% agreement (or disagreement) [40, 41]. Items were categorised as re-rate if they were: (1) changed due to qualitative feedback; (2) rated once and had 40–79% consensus; (3) rated twice with substantial alterations before the second rating and had a 40–79% consensus; (4) rated twice with minor alterations before the second rating, a 40–79% consensus, and > 5% change towards consensus. In Round 1, there were some inconsistencies between qualitative and quantitative responses (e.g., participants explicitly saying that they did not think the factor should be used and then rating ‘neither agree nor disagree’). These items were also re-administered in Round 2 alongside additional guidance to support participants with decision making. Items were categorised as ‘rejected’ if they: (1) had a consensus < 40%; (2) were rated twice with no alterations and had a consensus < 80%; (3) were rated twice with minor alterations, had a consensus < 80%, and < 5% change towards consensus. Following Round 3, final frequencies and consensus levels were calculated, as well as the mean score and standard deviation for each panel. The items in the final list were categorised as reaching consensus (≥ 80% rated disagree or agree), near-consensus (70–79% rated disagree or agree), or no consensus (< 70% rated disagree or agree). Items were grouped according to broader themes and the qualitative data coded to identify common rationales for ratings.

Analysis for the ranking task involved assigning a value of 10 (most important) to 1 (least important) to the items included in the list and a value of 0 to all other items. Mean rank was calculated for each item and used to signify the overall position of the item in the list. The percentage of participants who mentioned an item in their top 10 was also calculated and used to break ties when mean ranks were equal. Kendall’s coefficient of concordance (*W*) was calculated to evaluate the degree of consensus among respondents on the ranking task. The interpretation of *W* is as follows: weak < 0.3, moderate < 0.7, and strong ≥ 0.7. *W* was calculated using the ‘irr’ package in R programming software [45].

## Results

### Round 1–3: Delphi

The response rate per round, and the number of items rated/ranked, reached consensus/near consensus, re-rated, rejected, or new/modified per round are depicted in Fig. 1. The items that reached consensus for agreement or disagreement and their mean rating and level of consensus are outlined in Table 2. The full list of 87 statements, and their mean rating, and level of consensus are provided in Additional File 2.

**Table 2:** Patient prioritisation statements that reached consensus for agreement or disagreement and their mean rating and level of consensus

Items	Clinician				Lived experience			
Patients should be prioritised	Mean (*SD*)	Disagree (%)	Agree (%)	Consensus achieved	Mean (*SD*)	Disagree (%)	Agree (%)	Consensus achieved
Duration of Eating Disorder								
…if their eating disorder developed less than 6 months ago	**3.95 (0.83)**	**9%**	**82%**	**Yes**	3.14 (1.05)	22%	34%	No
…if their eating disorder developed less than 1 year ago	**4.00 (0.60)**	**7%**	**80%**	**Yes**	3.32 (0.91)	16%	46%	No
Body Weight and Behavioural Eating Disorder Symptoms								
Weight-related								
…if they are a very low weight	**4.25 (0.72)**	**2%**	**89%**	**Yes**	**3.93 (0.87)**	**9%**	**82%**	**Yes**
…if they are quickly losing weight (irrespective of their starting weight)	**4.30 (0.67)**	**2%**	**93%**	**Yes**	**4.18 (0.83)**	**5%**	**84%**	**Yes**
…if their weight is unstable (changing a lot) and they are underweight	**3.93 (0.70)**	**5%**	**89%**	**Yes**	3.86 (0.78)	8%	78%	Near
Compensatory								
…if they have reduced the amount or type of food they are eating (dietary restriction) at an extreme level (e.g., very little dietary intake almost every day)	**4.25 (0.62)**	**0%**	**91%**	**Yes**	**4.07 (0.76)**	**5%**	**86%**	**Yes**
…if they have diabetes and are purposefully restricting their insulin to lose weight (diabulimia)	**4.36 (0.75)**	**2%**	**89%**	**Yes**	**4.29 (0.80)**	**5%**	**89%**	**Yes**
Illness Severity								
…based upon the severity of their illness (taking into account psychological, physical, and social severity)	**4.50 (0.76)**	**2%**	**95%**	**Yes**	**4.13 (0.79)**	**5%**	**86%**	**Yes**
Individual Treatment Factors								
…if they have recently had treatment (within the last 6 months) but are now relapsing	**3.77 (0.57)**	**5%**	**80%**	**Yes**	3.84 (0.90)	11%	73%	Near
…if they are transitioning between child and adult services	**4.25 (0.69)**	**2%**	**91%**	**Yes**	3.68 (0.89)	10%	60%	No
…if they are transitioning between inpatient and community services	**4.27 (0.76)**	**5%**	**91%**	**Yes**	**4.20 (0.88)**	**4%**	**84%**	**Yes**
Service-related Factors								
…on a 'first-come first-serve' basis (people will receive treatment in the order in which they are referred, i.e., if Patient X’s referral arrived before Patient Y’s, Patient X will be seen first)	**2.05 (0.94)**	**80%**	**9%**	**Yes**^**a**^	2.61 (1.14)	55%	29%	No
…if their treatment was inappropriate, limited, or of poor quality (e.g., only re-feeding with limited therapeutic input)	3.63 (0.48)	0%	63%	No	**4.02 (0.87)**	**8%**	**80%**	**Yes**
…if they have been waiting a long time for treatment	**3.90 (0.59)**	**3%**	**83%**	**Yes**	**4.14 (0.73)**	**2%**	**84%**	**Yes**
Physical Health Factors								
…if they are at significant medical risk (e.g., very slow or irregular heartbeat, abnormal blood results)	**4.73 (0.49)**	**0%**	**98%**	**Yes**	**4.72 (0.64)**	**2%**	**93%**	**Yes**
…if their physical health is getting worse quickly (any metric of physical health)	**4.47 (0.74)**	**2%**	**98%**	**Yes**	**4.23 (0.81)**	**5%**	**93%**	**Yes**
…if they are experiencing medical problems because of their eating disorder (e.g., osteoporosis, fertility problems, bowel problems, problems with their heart or circulation)	**4.14 (0.79)**	**4%**	**84%**	**Yes**	**4.40 (0.72)**	**3%**	**93%**	**Yes**
…if they have a major physical disorder (e.g., cardiovascular disease, diabetes, cancer) that is made worse by their eating disorder	**4.20 (0.59)**	**0%**	**91%**	**Yes**	**4.07 (0.71)**	**5%**	**89%**	**Yes**
…if they are pregnant	**4.52 (0.58)**	**0%**	**96%**	**Yes**	**4.25 (0.82)**	**5%**	**87%**	**Yes**
…if they are experiencing malnutrition (as indicated by blood tests and irrespective of weight)	**4.27 (0.66)**	**0%**	**89%**	**Yes**	**4.18 (0.81)**	**5%**	**86%**	**Yes**
Mental Health Factors								
…if they are constantly having intrusive eating disorder related thoughts and feelings (e.g., thoughts about their body shape and weight, fear of putting on weight)	3.20 (0.88)	2%	48%	No	**4.14 (0.73)**	**2%**	**84%**	**Yes**
…if they are thinking or planning to end their life (suicide risk)	3.60 (1.05)	11%	55%	No	**4.30 (1.08)**	**9%**	**88%**	**Yes**
…if their mental health and well-being is getting worse quickly (any metric of mental health)	**4.09 (0.64)**	**0%**	**84%**	**Yes**	**4.29 (0.76)**	**4%**	**95%**	**Yes**
…if they have impaired or poor mental capacity/decision making because of their eating disorder	**4.23 (0.64)**	**0%**	**89%**	**Yes**	**4.11 (0.76)**	**4%**	**84%**	**Yes**
Life and Social Factors								
Individual Characteristics and Circumstances								
…if they are less than 12 years old	**4.23 (0.71)**	**2%**	**89%**	**Yes**	**4.30 (0.85)**	**5%**	**88%**	**Yes**
…if they are less than 18 years old	**3.98 (0.70)**	**5%**	**84%**	**Yes**	3.55 (0.89)	13%	59%	No
…if their eating disorder is negatively impacting their quality of life (e.g., stops them from doing leisure activities, impacts how they interact with other people or makes it difficult to work/study, financial problems)	3.68 (0.92)	15%	75%	Near	**4.14 (0.72)**	**4%**	**88%**	**Yes**

*Note.* Items in bold reached consensus. *SD* standard deviation

^a^ Consensus for disagreement

### Diagnosis

None of the ED diagnoses nor comorbid diagnoses reached consensus/near consensus in either panel. Common reasons for ratings were the belief that all ED diagnoses are equally serious and disruptive, and other factors, such as, impact on functioning, severity, and risk also needed to be considered. However, AN, bulimia nervosa (BN), and comorbidities were perceived by some respondents as elevating complexity and acute risk and therefore warranting prioritisation. Some perceived comorbidities as the responsibility of other services and/or requiring adapted treatment.

### Duration of eating disorder

An illness duration of < 6 months, < 1 year, and < 3 years reached consensus/near consensus for agreement in the clinician panel, but not the LE panel. Despite differences in ratings, qualitative comments were remarkably similar across the panels. There were numerous comments regarding the importance of early intervention for improving outcomes and increasing the likelihood of recovery. However, there were concerns regarding limited resources/capacity and the detrimental impact on individuals with longer illnesses (i.e., this group being deprioritised/excluded/given up on). Severity, risk, and willingness to engage were thought to take precedence over illness duration.

### Body weight and behavioural ED symptoms

For weight-related, binge eating, and compensatory ED symptoms, greater frequency/severity were associated with a higher level of agreement. Consensus was reached for very low weight, quickly losing weight (irrespective of starting weight), extreme dietary restriction, low and unstable weight, and if a diabetic patient was purposefully restricting/omitting their insulin. Any ED symptom in isolation, especially weight, was generally perceived as insufficient for priority setting. An understanding of severity, risk, distress, willingness to engage, and functioning were required for decision-making. Many were opposed to weight-based prioritisation as it can result in patients feeling they are ‘not sick enough’ to ‘deserve’ treatment.

### Illness severity

Overall severity considering psychological, physical, and social aspects reached consensus for agreement in both panels. It was important, particularly for LE experts, that severity incorporated all aspects of severity and not just physical or weight-related metrics. The dissensus for mild ED symptoms stems from the belief that intervening early will prevent worsening, but services do not have the capacity to do this and need to prioritise higher severity patients.

### Individual treatment factors

For items related to patients’ treatment history and responsiveness, consensus/near consensus was reached for prioritising patients who were relapsing after recent treatment or transitioning between inpatient and community, child and adult services, or to services in a different area. These were perceived as critical points in treatment where continuity of care is needed to prevent relapse and promote sustained recovery. Although the panels tended to disagree with prioritising those who had several rounds of previous treatment, there were comments on the need to not give up on this patient group. One item was removed after Round 1, as there were many comments about benefit from treatment being difficult, if not, impossible to objectively define, measure, or predict.

### Service-related factors

Service-related factors that reached consensus for agreement were waiting a long time for treatment in both panels and if the patient had received inappropriate, limited, or poor-quality care in the LE panel. Waiting a long time for treatment was perceived as detrimental for engagement and outcomes. Clinicians reached consensus for disagreement (i.e., to not use) for a ‘first-come first-served’ approach with a trend towards disagreeing in the LE panel. Participants felt that with resource constraints, patients should be prioritised according to severity, risk, and clinical need. Dissensus in prioritising patients who found it difficult to get a referral was due to the rating depending upon why the patient found it difficult.

### Physical health factors

All physical health-related items reached consensus for agreement across both panels. The items in this category had some of the highest levels of consensus. The high consensus was due to the imminent threat to health and life associated with these items and in the case of pregnancy, the risk to mother and baby.

### Mental health factors

Mental health getting worse quickly, impaired/poor cognitive capacity and decision-making, and high distress reached consensus/near consensus in both panels. Constantly having intrusive ED thoughts, suicide risk, and escalating non-suicidal self-injury reached consensus/near consensus in the LE panel. Some perceived intrusive ED thoughts as something experienced by all patients, and suicide risk and self-harm as the responsibility of other services. There were also frequent comments for the need to ensure that the mental health aspects of the ED should be considered equally important, if not more, than physical health. Motivation reached near consensus in the clinician panel as treatment can be more successful and shorter for motivated patients. However, others felt that lack of motivation is an indicator of severity, and that developing motivation is a key part of the treatment process.

### Life and social factors

Both panels reached consensus for agreement for prioritising patients < 12 years old, and clinicians reached consensus for patients < 18 years old. Early intervention to prevent the ED becoming entrenched/chronic/persistent and minimising the impact on the person’s development were the most frequently cited reasons for ratings. However, some felt that early intervention should be based on illness duration rather than age, and that younger patients already had separate services and better support systems. The ED negatively impacting quality of life reached consensus/near consensus for agreement in both panels. However, some felt that this item would apply to all patients and would therefore be difficult to prioritise. There was a trend towards disagreeing with prioritising based upon income, ethnicity, and if the patient was starting university soon, or had a small window of time before they moved. Having or living in a household with a high income reached near consensus for disagreement in both panels and only having a small window of time before they move somewhere else reached near consensus for disagreement in the LE panel. There were numerous comments on how ethnicity and income should not impact priority, and many felt that it was more important to support the patient in establishing care in the new area for university or a small window before they moved. Moreover, many felt that housing issues (e.g., homelessness) would need to be addressed before ED treatment. Of the social context items, having very little social support was the only item that reached near consensus for agreement. Social issues were perceived as increasing stress, but outside the remit of ED services.

### Round 4: Ranking

The results of the ranking task and *W* are outlined in Table 3 in rank order from most to least important. The ranks align closely with final consensus ratings. Medical risk, overall severity, and rapid physical deterioration were unanimously identified as the most important factors for priority setting in both panels. For clinicians, most of the other ‘top 10’ items were associated with heightened physiological risk (e.g., very low weight, rapidly losing weight), except for being < 12 years old and transitioning between inpatient and community. Clinicians commented on how physical risk needs to be addressed before psychological work can begin. Although physical risk items were prominent in the LE panel ‘top 10’, there was a greater emphasis on mental health factors, with items such as rapid mental deterioration, quality of life, suicide risk, and intrusive ED thoughts included. Participants with LE indicated that they felt uncomfortable placing physical risk items high on the list because mental health/emotional components are such an important and often neglected aspect of care that drives the ED, but also recognised that with limited resources physical risk needs to be a priority.

**Table 3:** Rank order of the top 10 items from most to least important

Clinician				Lived experience			
Rank and item	Mean rank (*SD*)	% Rated in top 10	% Consensus	Rank and item	Mean rank (*SD*)	% Rated in top 10	% Consensus
1.**if they are at significant medical risk (e.g., very slow or irregular heartbeat, abnormal blood results)**	**6.13 (3.96)**	**80%**	**98%**	1.**if they are at significant medical risk (e.g., very slow or irregular heartbeat, abnormal blood results)**	**6.17 (3.66)**	**81%**	**93%**
2.**based upon the severity of their illness (taking into account psychological, physical, and social severity)**	**4.13 (4.42)**	**56%**	**95%**	2.**based upon the severity of their illness (taking into account psychological, physical, and social severity)**	**5.98 (3.68)**	**83%**	**86%**
3.**if their physical health is getting worse quickly (any metric of physical health)**	**3.95 (4.08)**	**51%**	**98%**	3.**if their physical health is getting worse quickly (any metric of physical health)**	**3.38 (3.47)**	**63%**	**93%**
4.if they are pregnant	3.54 (3.24)	64%	96%	4.if their mental health and well-being is getting worse quickly (any metric of mental health)	3.31 (3.42)	60%	95%
5.if they have diabetes and are purposefully restricting their insulin to lose weight (diabulimia)	2.97 (3.20)	59%	89%	5.if they have a major physical disorder (e.g., cardiovascular disease, diabetes, cancer) that is made worse by their eating disorder	3.00 (3.31)	54%	89%
6.if they are transitioning between inpatient and community services	2.97 (3.50)	56%	91%	6.if their eating disorder is negatively impacting their quality of life (e.g., stops them from doing leisure activities, impacts how they interact with other people or makes it difficult to work/study, financial problems)	3.00 (3.59)	52%	88%
7.if they are quickly losing weight (irrespective of their starting weight)	2.69 (3.29)	46%	93%	7.if they are less than 12 years old	2.98 (3.52)	52%	88%
8.if they have reduced the amount or type of food they are eating (dietary restriction) at an extreme level (e.g., very little dietary intake almost every day)	2.54 (3.32)	41%	91%	8.if they are pregnant	2.90 (3.81)	44%	87%
9.if they are a very low weight	2.41 (3.27)	44%	89%	9.if they are thinking or planning to end their life (suicide risk)	2.85 (3.80)	42%	88%
10.if they are less than 12 years old	1.97 (3.07)	41%	89%	10.if they are constantly having intrusive eating disorder related thoughts and feelings (e.g., thoughts about their body shape and weight, fear of putting on weight)	2.69 (3.52)	54%	84%
	*W*	.14		*W*	.11		

*Note.* Items in bold obtained the same rank across participant groups. *SD* standard deviation, *W* Kendall’s coefficient or concordance

## Discussion

The aim of this study was to evaluate the degree of consensus and perceived relative importance of factors for priority setting decisions in ED services for two key stakeholder groups: clinicians and individuals with LE of an ED. To our knowledge, this is the first evaluation of clinician and LE opinions on priority setting in ED services. Despite differences in ‘expertise’, the pattern of responding was similar across the panels, with some notable differences. This is in accordance with previous Delphi studies in EDs and mental health more broadly, whereby consumers and professionals converge in their consensus [35, 40].

Medical risk, overall severity, and rapid physical deterioration were ranked as the top three factors for determining priority in clinician and LE panels. There was a strong view, particularly amongst LE participants, that severity should incorporate social and psychological aspects, not only physical. Most of the other items included in the ‘top 10’ for both panels were associated with a high degree of physical or mental risk (e.g., pregnancy, diabulimia, suicide). Clinicians included more physical risk and weight-related items, whereas the LE panel included mental health-related items, which were absent from the clinician’s ‘top 10’. Severity, physical health factors, and mental health risk items also obtained some of the highest levels of consensus across both panels. Moreover, consensus for weight and behavioural ED symptoms was greatest for the most severe/frequent and risky symptoms (e.g., vomiting 5 or more times per week). Qualitative comments also suggest that judgements throughout the study were largely influenced by the degree of risk or severity. Severity and acute risk are consistently identified as important for priority setting decisions in physical and mental healthcare and align with prioritarian principles of distributive justice. There appears to be a drive to treat those who are suffering the most or facing death [20, 32, 46]. These findings are consistent with ED clinical guidelines (i.e., 18) and practice, where the urgency of the patient’s condition tends to take precedence.

Some authors argue that preferentially allocating resources to those who are most unwell unjustly ignores those who will be worse later if left untreated, particularly when the most unwell will only benefit slightly [16]. The use of utilitarian principles such as this to justify choices were evident in the current study, albeit to a lesser extent than prioritarian (e.g., “*Early intervention is key, however, if another patient is deemed at greater physical & mental risk then this needs to be evaluated*”). This is in line with evidence demonstrating that people are generally willing to sacrifice some aggregate health gains to give priority to the most severely ill [20]. Utilitarian rationales were provided for many items, including transitions, age, illness duration, mild ED symptoms, and motivation.

Participants described transitions as poorly managed and crucial points where priority and continuity of care could promote sustained recovery and prevent relapse. The transition between inpatient and community services reached consensus in both panels and was included in the clinician’s ‘top 10’, underscoring its importance in priority setting. The transition between child and adult services and different areas reached consensus/near consensus in the clinician panel. Transitions have long been perceived as particularly challenging in EDs requiring careful co-ordination [47, 48]. The dangers of poorly managed transitions are evident in high profile cases, such as the death of 19-year-old Averil Hart in the UK [49].

An age of < 12 years old reached consensus and was included in the ‘top 10’ for both panels, suggesting a strong preference for prioritising the very young. Clinicians also reached consensus for patients < 18 years old. Comments suggest that younger patients were prioritised because of the belief that early intervention can lead to better outcomes and minimise the impact on development. This rationale did not hold as strongly for adolescents and emerging adults, despite evidence suggesting that a similar rationale may also be applicable to these age groups (e.g., [50, 51]). These findings largely align with recent efforts to ensure early access to ED treatment for children and young people [52] and broader healthcare priority setting literature, where younger patients tend to be prioritised for treatment [31, 32].

The consensus for illness duration items was notably different in clinician and LE panels. Only the clinician panel reached consensus/near consensus for prioritising patients with an illness duration of < 6 months, < 1 year, and < 3 years. The lack of endorsement of these items in the LE panel is likely due to concerns regarding the exclusion and neglect of patients with longer illness durations. Personal experiences of exclusion or difficulties accessing appropriate treatment may increase the strength of this concern in the LE panel. Indeed, clinical and research observations suggest that individuals with severe and enduring EDs are less likely to be in active ED treatment (for a myriad of reasons) [53]. Moreover, despite evidence in support of early intervention, predicting who will respond to what treatment and when, remains limited in EDs [54, 55]. Predictive uncertainty such as this makes the application of utilitarian principles difficult, leading to more egalitarian responses [56]. The LE panel appear to have a stronger preference for equity over utility in these circumstances. Conversely, clinical experience and observing the impact of early intervention on patients could strengthen ratings in the opposite direction. First Episode Rapid Early Intervention for EDs (FREED) is an early intervention service for emerging adults (16–25 years old) with recent onset EDs (< 3 years duration). FREED functions as a ‘service-within-a-service’, i.e., a smaller sub-group of clinicians in an evidence-based ED service are responsible for delivering FREED. FREED aims to reduce service-related delays to care and adapts evidence-based ED treatments to the needs of emerging adults in early-stage illness [23, 57]. Qualitative data gathered during the national scaling of FREED in England generally did not find that early intervention had a detrimental impact on non-FREED patients, if anything, the benefits were perceived as extending beyond FREED patients. Specifically, increased service efficiencies and the rapid response to treatment observed in FREED patients were seen as freeing up resources for non-FREED patients. Some of the materials and principles of FREED (e.g., attention to social media use) were also beneficial to non-FREED patients. Observing the impact of FREED on patients was noted as a key driver for using the model [Richards, Allen, & Schmidt, unpublished data]. Clinical experience of rationing treatment and considering the long-term implications of prioritisation decisions may also contribute towards the clinician preference for utility over equity.

The notably higher consensus for mental health and quality of life items in the LE panel and the inclusion of these items in the ‘top 10’ as well as the exclusion of weight-related items could, in part, stem from a drive to promote equity and parity of esteem between physical and mental health in ED services. There were numerous qualitative comments to support this: “*there needs to be equivalence of physical and mental symptoms*” or “*it should be based on the distress the person is experiencing and the impact it has on their life—NOT their weight*”. Mental health impacts were also described as the most problematic for patients and as the main driver of the ED. Physical health metrics, especially weight, have historically been used as one of the defining features of gaining access to ED services [58–60]. In recent years, there have been widespread campaigns (e.g., dump the scales [61]) and explicit instructions in clinical guidelines to not use weight or BMI as the only means of determining access treatment [18]. However, as this study and others demonstrate, the disparity between the physical and mental health components of the ED remains. More work is needed to consider the mental health and quality of life aspects of the ED in service access and priority setting.

Egalitarian principles were evident for diagnosis and broader life and social context. Many perceived all ED diagnoses as equally serious and debilitating, and that priority should be based on severity/risk/distress/impact on life rather than diagnosis. There was also limited endorsement of items related to the patient’s life and social context. Age, the impact of the ED on quality of life, and very limited social support were considered as pertinent factors. However, ethnicity, income, going to university, and a small window of time before moving somewhere else were deemed less relevant. Egalitarian rationales were provided for these items (i.e., individuals should not be disadvantaged by personal circumstances) and parallel findings in the wider priority setting literature [31, 32]. However, a “pure” equity approach was not sought. The ‘first-come first-served’ method was the only item to reach consensus for disagreement (i.e., should not be used). The blindness of this approach to factors that would be inappropriate to ignore (e.g., medical risk) led to the rejection of this item. Participants did however comment on how they wished that priority could be determined in this way (e.g., “*Whilst I would like this to be the case there will be some people who are more urgently in need*”).

### Clinical implications

One of the key findings of this study is the greater emphasis on mental health symptoms and quality of life in the LE panel. There were strong opinions against prioritising solely based upon physical metrics, especially weight-related criteria. Physical risk is currently one of the main prioritisation factors used in services, and as pressure escalates, the focus on physical risk becomes greater. It is important to raise awareness of and address this over-reliance on physical metrics within ED services. Given current pressures on services, prioritising based upon anything else can feel like a luxury, however, these findings indicate that this is not a luxury and that the whole person needs to be kept in mind as much as possible. The development of an ED prioritisation tool to facilitate discussions around priority setting and to ensure that all aspects of the person are considered could help address this imbalance. Prioritisation tools incorporating measures of risk, symptoms, psychosocial functioning, and the impact on the person’s life have been used to promote transparent and equitable priority setting in other areas of healthcare (e.g., [62]). There will be a degree of subjectivity in quantifying certain metrics (e.g., quality of life), as every patient is different, and some may lack insight into their ED symptoms and the impact of these on their daily functioning. However, this does not necessarily mean that these features cannot be meaningfully considered alongside other metrics to inform patient priority decisions. Such prioritisation tools could also be used as an indicator for the condition of services (e.g., the discrepancy between demand and capacity) and stimulate discussions with service commissioners and policy makers around adequately funding services.

Another important discrepancy between clinician and LE opinions was the greater endorsement of prioritising patients in early-stage illness in the clinician panel. Participants with LE did not perceive patients in early-stage illness as a priority, largely because they did not want other patient groups to be disadvantaged. To be considered as a priority, early intervention therefore needs to be adequately resourced to ensure that it does not negatively impact the care of others. In addition to effective priority setting procedures, there is also a pressing need to address capacity issues and pressures on specialist ED services. Promising avenues to relieve pressure on services include increasing the reach of effective prevention programs [63], implementing task-sharing interventions (e.g., peer support, guided self-help) [64], and more initiatives for the early identification and treatment of EDs in educational and primary care settings [65, 66].

### Strengths and limitations

A major strength of this study was that we were able to recruit a large sample with high retention across the rounds. Participants also provided detailed responses to the optional comment boxes and open-ended questions, which provided insights into why people gave specific ratings and increased the validity of our conclusions. The inclusion of both clinician and LE opinions from across the UK was another strength of this study. However, there are several limitations that need to be considered. First, the recruitment method, i.e., self-selection and largely through social media, may have introduced a bias in the sample. Only those who were motivated and active on social media would have the opportunity to participate. Participant motivation for taking part was not assessed and may have biased the results. Diagnoses and recovery/illness status were also not verified with standardised criteria or clinical interviews and may have impacted how participants responded to the questionnaires. Additionally, while the inclusion criteria were deliberately broad to increase diversity of experiences, one year’s worth of experience in EDs may not have been sufficient to develop clinical ‘expertise’ in this area. Moreover, although the sample was diverse in some respects (e.g., profession), it was not in others (e.g., ethnicity). Caution is therefore needed when generalising these findings, particularly for items that relate to under-represented characteristics. Second, as comment boxes and open-ended questions were optional, not everyone provided a rationale for their choice. This makes the qualitative data on why participants chose certain options “incomplete”. Finally, participants expressed difficulties in rating and ranking items. Prioritisation decisions are highly complex and difficult, and single item ratings vastly underestimate this complexity. In practice, decisions are rarely made on a single factor and dimensions of the decision-making process were not included in this study. For example, one issue, which was raised by participants in their qualitative feedback, was the lack of specification on precisely what type of intervention or care the patient was being prioritised for. Decision making is likely to differ for prioritising patients for physical monitoring/observations/care versus psychosocial interventions. There was also overlap between factors which complicated the decision making in the ranking task. While this study provides an important starting point for discussions around priority setting in EDs, more research is needed utilising more ecologically valid techniques. Additionally, an in-depth ethnographic study using observations of priority setting behaviour alongside interviews with clinicians and patients would be a useful addition to this evidence-base.

## Conclusions

Priority setting decisions are ethically complex, difficult, and can have considerable consequences for those involved. Yet, research to guide discussions and support clinical decision making in ED services is absent. EDs are unique as they carry considerable physical and psychological risks that need to be considered during priority setting decisions. Our findings demonstrate that clinicians and individuals with LE place physical and psychological risk and severity (prioritarianism) at the top of determining priority in ED services. Followed by a mix of utilitarian and egalitarian approaches with clinicians placing greater emphasis on the former and individuals with LE on the latter. While further testing of these findings is warranted in more heterogenous samples and with more ecologically valid designs, we hope that this paper will stimulate discussion for this important topic. Now more than ever, there is a pressing need for research to support conversations regarding fair, just, and transparent priority setting in EDs.

## Supplementary Information

**Additional file 1. **Round 1 questionnaire development. 

**Additional file 2: Table 1. **Fulllist of all patient prioritisation statements and their mean rating and levelof consensus. 

## Data Availability

The datasets generated and/or analysed during the current study are not publicly available due to the conditions of consent and to protect the anonymity of participants but are available from the corresponding author on reasonable request.

## Abbreviations

AEDS: Adult eating disorder service
AMHS: Adult mental health service
AN: Anorexia nervosa
ARFID: Avoidant/restrictive food intake disorder
BMI: Body mass index
BN: Bulimia nervosa
CAEDS: Child and adolescent eating disorder service
CAMHS: Child and adolescent mental health service
ED: Eating disorder
FREED: First Episode Rapid Early Intervention for Eating Disorders
LE: Lived experience
NHS: National Health Service
OCD: Obsessive compulsive disorder
