# Supplementary material for: A Delphi study to explore clinician and lived experience perspectives on setting priorities in eating disorder services
Source: BMC Health Serv Res. 2022 Jun 17;22:788. doi: 10.1186/s12913-022-08170-4 (PMC9206284; doi:10.1186/s12913-022-08170-4)
Supplement: Supplementary file 1 — Additional file 1. Round 1 questionnaire development. [file 12913_2022_8170_MOESM1_ESM.docx]

# Supplementary Materials

**Round 1 Questionnaire Development**

The Round 1 questionnaire was developed by conducting a systematic literature review followed by consultation and pre-testing with eating disorder (ED) clinicians and individuals with a lived experience (LE). For the literature review, PsychInfo and Medline databases were searched on 24th August 2020 using the following search terms: (eating disorder OR anorexi* OR bulimi* OR binge eat*) AND ((allocation AND resource*) OR priorit*). The search yielded 288 unique articles with only one article being relevant for the current study. The included article was Gajre, McClelland, and Furnham (2018) study. This study involved a survey in which 361 participants were given a list of eight hypothetical patients with either obesity or anorexia nervosa (AN). The patients varied on three factors: age, social class, and mental health history. Participants were required to choose which patient should receive treatment and then rank patients in order of priority. Age, social class, and comorbid mental health diagnoses were all found to impact priority. Patients with obesity or AN were more likely to be prioritised if they were younger, with a comorbid mental health problem and from a low social class. Following the literature search, KR (lead author) drafted a list of clinical and non-clinical factors for patient prioritisation. This list was then distributed amongst clinicians and researchers in the ED unit at the King’s College London/South London and Maudsley Hospital for feedback on appropriateness and wording, and for suggested new items. Clinician and LE versions of the questionnaire were then drafted. These draft questionnaires were given to five clinicians and five individuals with a LE for additional feedback on content, formatting, and language, and suggested new items. The final questionnaire for Round 1 consisted of demographic questions (e.g., age, gender, profession), 49 items for prioritising patients within ED services, and open-ended questions for feedback and suggested new items. A copy of the questionnaire can be provided on request of the authors.
