# Supplementary material for: A Delphi study to explore clinician and lived experience perspectives on setting priorities in eating disorder services
Source: BMC Health Serv Res. 2022 Jun 17;22:788. doi: 10.1186/s12913-022-08170-4 (PMC9206284; doi:10.1186/s12913-022-08170-4)
Supplement: Supplementary file 2 — Additional file 2: Table 1. Fulllist of all patient prioritisation statements and their mean rating and levelof consensus. [file 12913_2022_8170_MOESM2_ESM.docx]

Supplementary Materials

Table 1. Full list of all patient prioritisation statements and their mean rating and level of consensus.

| Items | Clinician | | | | Lived experience | | | |
| --- | --- | --- | --- | --- | --- | --- | --- | --- |
| Patients should be prioritised…. | Mean (*SD*) | Disagree (%) | Agree (%) | Consensus achieved | Mean (*SD*) | Disagree (%) | Agree (%) | Consensus achieved |
| Diagnostic Factors |  |  |  |  |  |  |  |  |
| Eating disorder diagnosis |  |  |  |  |  |  |  |  |
| …if they have a diagnosis of anorexia nervosa | 3.48 (0.86) | 14% | 50% | No | 2.93 (0.83) | 29% | 18% | No |
| …if they have a diagnosis of bulimia nervosa | 2.91 (0.83) | 36% | 25% | No | 2.91 (0.75) | 27% | 13% | No |
| …if they have a diagnosis of binge eating disorder | 2.55 (0.70) | 48% | 7% | No | 2.84 (0.73) | 29% | 11% | No |
| …if they have a diagnosis of other specified feeding or eating disorder | 2.68 (0.60) | 36% | 2% | No | 2.89 (0.68) | 27% | 4% | No |
| …if they have a diagnosis of avoidant restrictive food intake disorder (ARFID) | 2.66 (0.65) | 39% | 7% | No | 2.88 (0.72) | 30% | 16% | No |
| Comorbid diagnosis |  |  |  |  |  |  |  |  |
| …if they have an intellectual disability | 3.02 (0.66) | 21% | 23% | No | 2.80 (0.75) | 38% | 16% | No |
| …if they are also experiencing a mild mood, anxiety, or stress-related disorder (e.g., depression, bipolar disorder, social phobia, panic disorder, post-traumatic stress disorder) | 2.58 (0.74) | 48% | 10% | No | 2.91 (0.82) | 36% | 25% | No |
| …if they are also experiencing a moderate mood, anxiety, or stress-related disorder (e.g., depression, bipolar disorder, social phobia, panic disorder, post-traumatic stress disorder) | 2.95 (0.86) | 32% | 27% | No | 3.17 (0.83) | 28% | 42% | No |
| …if they are also experiencing a severe mood, anxiety, or stress-related disorder (e.g., depression, bipolar disorder, social phobia, panic disorder, post-traumatic stress disorder) | 3.38 (0.95) | 3% | 48% | No | 3.76 (1.02) | 16% | 66% | No |
| …if they are also abusing or are dependent on alcohol or other drugs (substance use disorder) | 2.52 (0.73) | 48% | 7% | No | 2.79 (0.91) | 43% | 22% | No |
| …if they also have an autism spectrum disorder | 2.95 (0.75) | 27% | 21% | No | 2.93 (0.85) | 36% | 25% | No |
| …if they also have a personality disorder | 2.52 (0.73) | 43% | 5% | No | 2.50 (0.60) | 48% | 2% | No |
| …if they are also experiencing an obsessive-compulsive or related disorder (e.g., OCD, body dysmorphic disorder) | 2.77 (0.68) | 36% | 14% | No | 3.07 (0.88) | 25% | 27% | No |
|  |  |  |  |  |  |  |  |  |
| Duration of Eating Disorder |  |  |  |  |  |  |  |  |
| …if their eating disorder developed less than 6 months ago | **3.95 (0.83)** | **9%** | **82%** | **Yes** | 3.14 (1.05) | 22% | 34% | No |
| …if their eating disorder developed less than 1 year ago | **4.00 (0.60)** | **7%** | **80%** | **Yes** | 3.32 (0.91) | 16% | 46% | No |
| …if their eating disorder developed less than 3 years ago | 3.83 (0.74) | 5% | 73% | Near | 3.21 (0.83) | 16% | 39% | No |
| …if they have had an eating disorder for 5 years or more | 2.80 (0.59) | 30% | 9% | No | 3.54 (0.89) | 14% | 56% | No |
| …if they have had an eating disorder for 10 years or more | 2.66 (0.58) | 39% | 5% | No | 3.68 (0.99) | 14% | 58% | No |
| …if they have had an eating disorder for 15 years or more | 2.59 (0.62) | 38% | 2% | No | 3.66 (1.00) | 14% | 56% | No |
|  |  |  |  |  |  |  |  |  |
| Body Weight and Behavioural Eating Disorder Symptoms |  |  |  |  |  |  |  |  |
| Weight-related |  |  |  |  |  |  |  |  |
| …if they are underweight (this includes all levels of being underweight) | 3.53 (0.85) | 10% | 58% | No | 3.18 (0.96) | 23% | 38% | No |
| …if they are a very low weight | **4.25 (0.72)** | **2%** | **89%** | **Yes** | **3.93 (0.87)** | **9%** | **82%** | **Yes** |
| …if they are experiencing obesity and an eating disorder | 2.58 (0.78) | 45% | 10% | No | 2.86 (0.82) | 38% | 30% | No |
| …if they are experiencing morbid obesity and an eating disorder | 2.93 (0.87) | 32% | 30% | No | 3.27 (0.90) | 21% | 39% | No |
| …if they are quickly losing weight (irrespective of their starting weight) | **4.30 (0.67)** | **2%** | **93%** | **Yes** | **4.18 (0.83)** | **5%** | **84%** | **Yes** |
| …if their weight is stable and they are underweight | 2.80 (0.80) | 39% | 21% | No | 2.86 (0.75) | 34% | 18% | No |
| …if their weight is stable and they are neither under- nor overweight | 2.17 (0.71) | 65% | 0% | No | 2.49 (0.61) | 45% | 0% | No |
| …if their weight is stable and they are overweight | 2.25 (0.67) | 63% | 0% | No | 2.51 (0.61) | 47% | 2% | No |
| …if their weight is unstable (changing a lot) and they are underweight | **3.93 (0.70)** | **5%** | **89%** | **Yes** | 3.86 (0.78) | 8% | 78% | Near |
| …if their weight is unstable (changing a lot) and they are neither under- nor overweight | 3.09 (0.74) | 21% | 27% | No | 3.54 (0.84) | 12% | 62% | No |
| …if their weight is unstable (changing a lot) and they are overweight | 2.95 (0.89) | 27% | 30% | No | 3.40 (0.83) | 18% | 54% | No |
| Binge Eating |  |  |  |  |  |  |  |  |
| …if they are binge eating once a week or less | 2.08 (0.66) | 75% | 0% | Near^a^ | 2.28 (0.73) | 64% | 4% | No |
| …if they are binge eating 2-4 times a week | 2.65 (0.89) | 43% | 18% | No | 3.20 (0.84) | 20% | 32% | No |
| …if they are binge eating 5 times or more per week | 3.18 (0.97) | 23% | 39% | No | 3.62 (.92) | 14% | 60% | No |
| Compensatory |  |  |  |  |  |  |  |  |
| …if they are exercising excessively/compulsively | 3.33 (0.64) | 7% | 37% | No | 3.56 (0.71) | 12% | 68% | No |
| …if they are making themselves vomit once a week or less | 2.28 (0.64) | 63% | 0% | No | 2.62 (0.67) | 48% | 10% | No |
| …if they are making themselves vomit 2-4 times per week | 3.11 (0.90) | 23% | 36% | No | 3.41 (0.81) | 16% | 53% | No |
| …if they are making themselves vomit 5 times or more per week | 3.95 (0.69) | 3% | 79% | Near | 4.02 (0.94) | 2% | 76% | Near |
| …if they are abusing laxatives or diuretics once a week or less | 2.48 (0.76) | 48% | 5% | No | 2.60 (0.61) | 42% | 4% | No |
| …if they are abusing laxatives or diuretics 2-4 times per week | 3.03 (0.86) | 25% | 32% | No | 3.33 (0.66) | 8% | 39% | No |
| …if they are abusing laxatives or diuretics 5 times or more per week | 3.65 (0.83) | 8% | 65% | No | 3.88 (0.75) | 4% | 74% | Near |
| …if they have reduced the amount or type of food they are eating (dietary restriction) at a mild to moderate level (e.g., restricting on some days and not others, or restriction of a specific food group) | 2.55 (0.55) | 48% | 3% | No | 2.82 (0.80) | 38% | 22% | No |
| …if they have reduced the amount or type of food they are eating (dietary restriction) at an extreme level (e.g., very little dietary intake almost every day) | **4.25 (0.62)** | **0%** | **91%** | **Yes** | **4.07 (0.76)** | **5%** | **86%** | **Yes** |
| …if they have diabetes and are purposefully restricting their insulin to lose weight (diabulimia) | **4.36 (0.75)** | **2%** | **89%** | **Yes** | **4.29 (0.80)** | **5%** | **89%** | **Yes** |
|  |  |  |  |  |  |  |  |  |
| Illness Severity |  |  |  |  |  |  |  |  |
| …if they are experiencing mild eating disorder symptoms (e.g., weight/shape concerns, infrequent binge eating or fasting) | 2.78 (0.95) | 36% | 22% | No | 2.92 (1.01) | 37% | 25% | No |
| …based upon the severity of their illness (taking into account psychological, physical, and social severity) | **4.50 (0.76)** | **2%** | **95%** | **Yes** | **4.13 (0.79)** | **5%** | **86%** | **Yes** |
|  |  |  |  |  |  |  |  |  |
| Individual Treatment Factors |  |  |  |  |  |  |  |  |
| …if they have had several rounds of previous eating disorder treatment | 2.40 (0.78) | 60% | 5% | No | 2.54 (0.65) | 46% | 4% | No |
| …if they have not accessed eating disorder services before | 3.06 (0.88) | 29% | 29% | No | 3.08 (1.12) | 33% | 32% | No |
| …if they have recently had treatment (within the last 6 months) but are now relapsing | **3.77 (0.57)** | **5%** | **80%** | **Yes** | 3.84 (0.90) | 11% | 73% | Near |
| …if they are receiving treatment from another public mental health service | 2.61 (0.49) | 39% | 0% | No | 2.42 (0.73) | 60% | 8% | No |
| …if they are transitioning between child and adult services | **4.25 (0.69)** | **2%** | **91%** | **Yes** | 3.68 (0.89) | 10% | 60% | No |
| …if they are transitioning between inpatient and community services | **4.27 (0.76)** | **5%** | **91%** | **Yes** | **4.20 (0.88)** | **4%** | **84%** | **Yes** |
| …if they are transitioning between services in different areas | 3.90 (0.80) | 5% | 78% | Near | 3.60 (0.86) | 36% | 64% | No |
| …based upon how much they are likely to benefit from treatment | 3.00 (1.10) | 33% | 37% | No | 3.02 (1.28) | 37% | 40% | No |
|  |  |  |  |  |  |  |  |  |
| Service-related Factors |  |  |  |  |  |  |  |  |
| …on a 'first-come first-serve' basis (people will receive treatment in the order in which they are referred, i.e., if Patient X’s referral arrived before Patient Y’s, Patient X will be seen first) | **2.05 (0.94)** | **80%** | **9%** | **Yes^a^** | 2.61 (1.14) | 55% | 29% | No |
| …if they found it difficult to get a referral to the eating disorder service (possible reasons for difficulties include lack of recognition, internal delays between services, and referrals being missed) | 2.95 (0.75) | 27% | 21% | No | 3.20 (0.83) | 18% | 33% | No |
| …if their treatment was inappropriate, limited, or of poor quality (e.g., only re-feeding with limited therapeutic input) | 3.63 (0.48) | 0% | 63% | No | **4.02 (0.87)** | **8%** | **80%** | **Yes** |
| …if they do not have access to specialist eating disorder care within their area (i.e., have to be sent out of area for treatment) | 3.16 (0.65) | 9% | 27% | No | 3.28 (0.78) | 16% | 46% | No |
| …if they have been waiting a long time for treatment | **3.90 (0.59)** | **3%** | **83%** | **Yes** | **4.14 (0.73)** | **2%** | **84%** | **Yes** |
|  |  |  |  |  |  |  |  |  |
| Physical Health Factors |  |  |  |  |  |  |  |  |
| …if they are at significant medical risk (e.g., very slow or irregular heartbeat, abnormal blood results) | **4.73 (0.49)** | **0%** | **98%** | **Yes** | **4.72 (0.64)** | **2%** | **93%** | **Yes** |
| …if their physical health is getting worse quickly (any metric of physical health) | **4.47 (0.74)** | **2%** | **98%** | **Yes** | **4.23 (0.81)** | **5%** | **93%** | **Yes** |
| …if they are experiencing medical problems because of their eating disorder (e.g., osteoporosis, fertility problems, bowel problems, problems with their heart or circulation) | **4.14 (0.79)** | **4%** | **84%** | **Yes** | **4.40 (0.72)** | **3%** | **93%** | **Yes** |
| …if they have a major physical disorder (e.g., cardiovascular disease, diabetes, cancer) that is made worse by their eating disorder | **4.20 (0.59)** | **0%** | **91%** | **Yes** | **4.07 (0.71)** | **5%** | **89%** | **Yes** |
| …if they are pregnant | **4.52 (0.58)** | **0%** | **96%** | **Yes** | **4.25 (0.82)** | **5%** | **87%** | **Yes** |
| …if they are experiencing malnutrition (as indicated by blood tests and irrespective of weight) | **4.27 (0.66)** | **0%** | **89%** | **Yes** | **4.18 (0.81)** | **5%** | **86%** | **Yes** |
|  |  |  |  |  |  |  |  |  |
| Mental Health Factors |  |  |  |  |  |  |  |  |
| …if they are constantly having intrusive eating disorder related thoughts and feelings (e.g., thoughts about their body shape and weight, fear of putting on weight) | 3.20 (0.88) | 2% | 48% | No | **4.14 (0.73)** | **2%** | **84%** | **Yes** |
| …if they are thinking or planning to end their life (suicide risk) | 3.60 (1.05) | 11% | 55% | No | **4.30 (1.08)** | **9%** | **88%** | **Yes** |
| …if they have escalating non-suicidal self-injury behaviours (i.e., becoming more intense or frequent) | 2.89 (0.87) | 34% | 27% | No | 3.61 (0.94) | 18% | 71% | Near |
| …if they have stable non-suicidal self-injury behaviours (i.e., has not changed in frequency or presentation for a while) | 2.53 (0.55) | 50% | 3% | No | 2.98 (0.84) | 30% | 29% | No |
| …if their mental health and well-being is getting worse quickly (any metric of mental health) | **4.09 (0.64)** | **0%** | **84%** | **Yes** | **4.29 (0.76)** | **4%** | **95%** | **Yes** |
| …if they are highly distressed by their eating disorder | 3.65 (0.66) | 8% | 70% | Near | 4.24 (0.85) | 2% | 78% | Near |
| …if they have impaired or poor mental capacity/decision making because of their eating disorder | **4.23 (0.64)** | **0%** | **89%** | **Yes** | **4.11 (0.76)** | **4%** | **84%** | **Yes** |
| …if they are motivated for treatment or to get better | 3.86 (0.98) | 14% | 73% | Near | 3.41 (1.16) | 23% | 25% | No |
|  |  |  |  |  |  |  |  |  |
| Life and Social Factors |  |  |  |  |  |  |  |  |
| Individual Characteristics and Circumstances |  |  |  |  |  |  |  |  |
| …if they are less than 12 years old | **4.23 (0.71)** | **2%** | **89%** | **Yes** | **4.30 (0.85)** | **5%** | **88%** | **Yes** |
| …if they are less than 18 years old | **3.98 (0.70)** | **5%** | **84%** | **Yes** | 3.55 (0.89) | 13% | 59% | No |
| …if they are less than 25 years old | 3.59 (0.73) | 7% | 59% | No | 3.00 (0.99) | 27% | 30% | No |
| …if they are a member of an ethnic minority group | 2.68 (0.57) | 38% | 5% | No | 2.62 (0.73) | 44% | 10% | No |
| …if they are starting university soon | 3.00 (0.75) | 25% | 23% | No | 2.72 (0.90) | 40% | 18% | No |
| …if they only have a small window of time before they move somewhere else | 2.66 (0.75) | 46% | 14% | No | 2.27 (0.67) | 73% | 4% | Near^a^ |
| …if their eating disorder is negatively impacting their quality of life (e.g., stops them from doing leisure activities, impacts how they interact with other people or makes it difficult to work/study, financial problems) | 3.68 (0.92) | 15% | 75% | Near | **4.14 (0.72)** | **4%** | **88%** | **Yes** |
| …if they have or do live in a household with a low income | 2.52 (0.76) | 50% | 9% | No | 2.84 (0.95) | 36% | 23% | No |
| …if they are homeless or do not have secure housing | 2.89 (0.90) | 32% | 27% | No | 3.25 (1.05) | 29% | 38% | No |
| …if they have or do live in a household with a high income | 1.68 (0.83) | 77% | 0% | Near^a^ | 1.73 (0.80) | 79% | 0% | Near^a^ |
| Social Context |  |  |  |  |  |  |  |  |
| …if they are a parent or have a child that depends on them or are the main carer for an elderly relative | 3.63 (0.49) | 0% | 63% | No | 3.55 (0.88) | 14% | 57% | No |
| …if they have very little social support (i.e., are isolated, have very little support or contact/interaction with others) | 3.68 (0.66) | 8% | 73% | Near | 3.60 (0.94) | 16% | 68% | No |
| …if they are at-risk of harm from others | 3.25 (0.69) | 14% | 39% | No | 3.32 (0.97) | 26% | 39% | No |
| …if another member of their family is receiving treatment for an eating disorder | 2.93 (0.82) | 27% | 25% | No | 2.70 (0.81) | 40% | 16% | No |
| …if another member of their family is receiving treatment for any other mental health problem | 2.82 (0.76) | 30% | 16% | No | 2.38 (0.73) | 60% | 6% | No |
| …if the person's carer/family/friends/significant other is experiencing a high level of fatigue and stress (related to supporting the person with the eating disorder) | 3.11 (0.72) | 21% | 32% | No | 3.00 (0.95) | 29% | 29% | No |

*Note.* Items in bold reached consensus. SD = standard deviation; OCD = obsessive compulsive disorder.

^a^Consensus or near consensus for disagreement.
